# Supplementary material for: The interactome of intact mitochondria by cross-linking mass spectrometry provides evidence for coexisting respiratory supercomplexes
Source: Mol Cell Proteomics. 2017 Dec 8;17(2):216–32. doi: 10.1074/mcp.RA117.000470 (PMC5795388; doi:10.1074/mcp.RA117.000470)
Supplement: Supplemental Data [file supp_RA117.000470_133922_0_supp_23978_fzffwf.zip › spectra_annotation/mito_spectra_annotation/282-1-4-1-4-1.pdf]

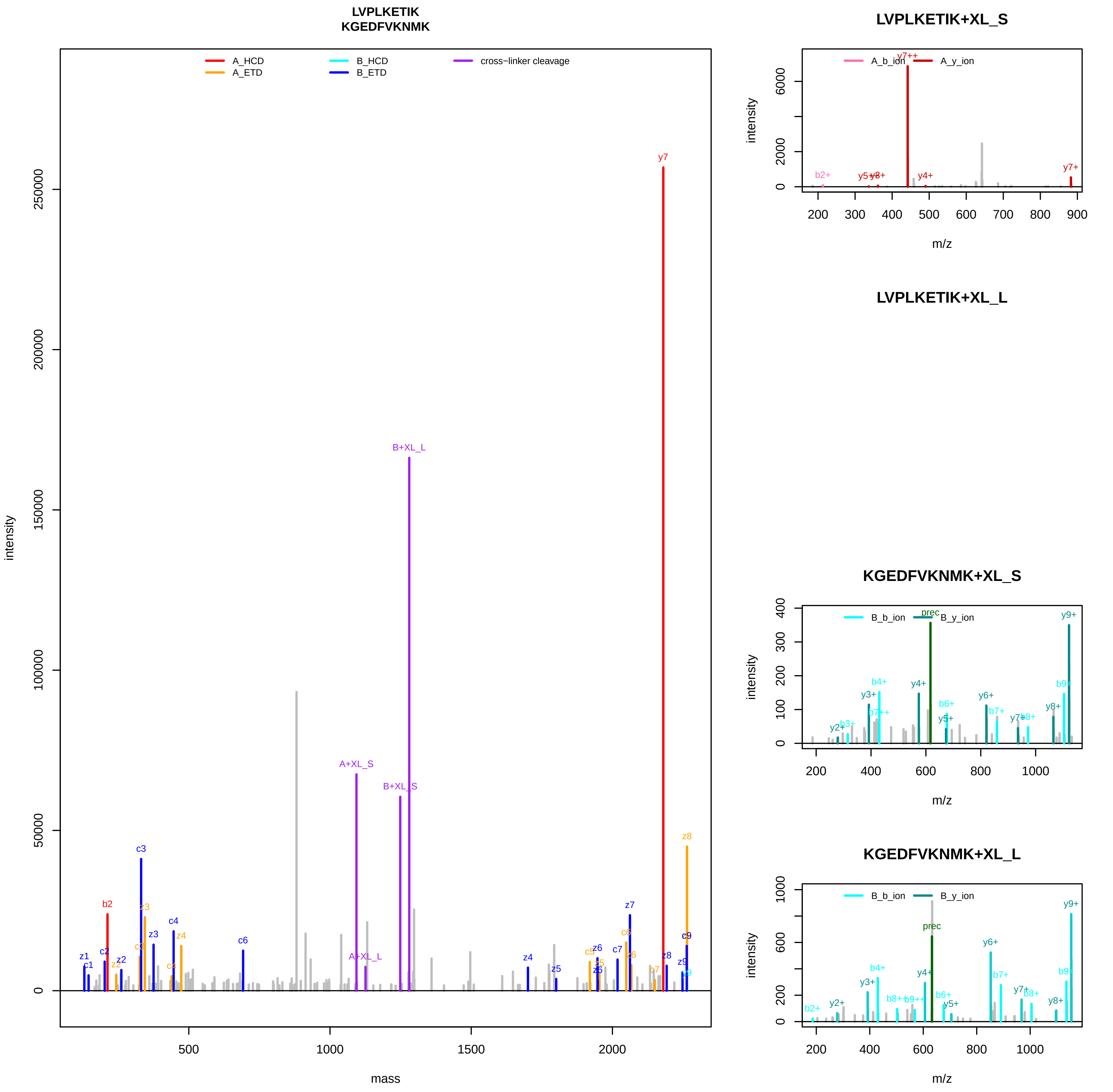

LVPLKETIK
KGEDFVKNMK
A_HCD
A_ETD
B_HCD
B_ETD
cross−linker cleavage
intensity
mass
0
50000
100000
150000
200000
250000
500
1000
1500
2000
y7
B+XL_L
A+XL_S
B+XL_S
A+XL_L
z8
c3
b2
z3
c4
z3
z4
c6
z4
z5
c5
z6
c7
z7
c6
z8
c9
z9
c7
z1
c1
c2
z2
z2
c3
c4
LVPLKETIK+XL_S
A_b_ion
A_y_ion
y7++
b2+
y5+
y8+
y4+
y7+
m/z
0
2000
6000
200
300
400
500
600
700
800
900
LVPLKETIK+XL_L
KGEDFVKNMK+XL_S
B_b_ion
B_y_ion
prec
y9+
b4+
y4+
y3+
b6+
y6+
b7++
b7+
y2+
b3+
y5+
y7+
b8+
y8+
b9+
0
100
200
300
400
200
400
600
800
1000
KGEDFVKNMK+XL_L
B_b_ion
B_y_ion
y9+
prec
y6+
b4+
y3+
y4+
b7+
b8+
b9++
b6+
y5+
b2+
y2+
y7+
b8+
y8+
b9+
0
200
600
1000
200
400
600
800
1000
